# Multi-omic profiling reveals discrepant immunogenic properties and a unique tumor microenvironment among melanoma brain metastases

**DOI:** 10.1038/s41698-023-00471-z

**Published:** 2023-11-14

**Authors:** Gino K. In, Jennifer R. Ribeiro, Jun Yin, Joanne Xiu, Matias A. Bustos, Fumito Ito, Frances Chow, Gabriel Zada, Lindsay Hwang, April K. S. Salama, Soo J. Park, Justin C. Moser, Sourat Darabi, Evidio Domingo-Musibay, Maria L. Ascierto, Kim Margolin, Jose Lutzky, Geoffrey T. Gibney, Michael B. Atkins, Benjamin Izar, Dave S. B. Hoon, Ari M. VanderWalde

**Affiliations:** 1 Division of Oncology, Norris Comprehensive Cancer Center, Keck School of Medicine, University of Southern California, Los Angeles, CA USA; 2 Caris Life Sciences, Phoenix, AZ USA; 3 Department of Translational Molecular Medicine, Saint John’s Cancer Institute, Providence Saint John’s Health Center, Santa Monica, CA USA; 4 Department of Surgery, Norris Comprehensive Cancer Center, Keck School of Medicine, University of Southern California, Los Angeles, CA USA; 5 Department of Neurology, Norris Comprehensive Cancer Center, Keck School of Medicine, University of Southern California, Los Angeles, CA USA; 6 Department of Neurological Surgery, Norris Comprehensive Cancer Center, Keck School of Medicine, University of Southern California, Los Angeles, CA USA; 7 LAC+USC Medical Center, Los Angeles, CA USA; 8 Department of Radiation Oncology, Norris Comprehensive Cancer Center, Keck School of Medicine, University of Southern California, Los Angeles, CA USA; 9 Division of Medical Oncology, Duke Cancer Institute, Duke University, Durham, NC USA; 10 Division of Hematology/Oncology, Moores Cancer Center, University of California San Diego, La Jolla, CA USA; 11 HonorHealth Research and Innovation Institute, Scottsdale, AZ USA; 12 Hoag Family Cancer Institute, Hoag Hospital, Newport Beach, CA USA; 13 Department of Medicine, Masonic Cancer Center, University of Minnesota Medical School, Minneapolis, MN USA; 14 Rosalie and Harold Rae Brown Cancer Immunotherapy Research Program, Borstein Family Melanoma Program, Department of Translational Immunology, Saint John’s Cancer Institute, Providence Saint John’s Health Center, Santa Monica, CA USA; 15 Department of Medical Oncology, Saint John’s Cancer Institute, Providence Saint John’s Health Center, Santa Monica, CA USA; 16 Sylvester Comprehensive Cancer Center, University of Miami Health System, Miami, FL USA; 17 Division of Hematology and Oncology, Lombardi Comprehensive Cancer Center, Georgetown University Hospital, Washington, DC USA; 18 Georgetown-Lombardi Comprehensive Cancer Center, Washington, DC USA; 19 Columbia University, Herbert Irving Comprehensive Cancer Center, New York, NY USA; 20 Caris Life Sciences, Irving, TX USA; 21 West Cancer Center and Research Institute, 514 Chickasawba St., Blytheville, Arkansas 72315 USA

**Keywords:** Melanoma, Cancer genomics, Cancer microenvironment

## Abstract

Melanoma brain metastases (MBM) are clinically challenging to treat and exhibit variable responses to immune checkpoint therapies. Prior research suggests that MBM exhibit poor tumor immune responses and are enriched in oxidative phosphorylation. Here, we report results from a multi-omic analysis of a large, real-world melanoma cohort. MBM exhibited lower interferon-gamma (IFNγ) scores and T cell-inflamed scores compared to primary cutaneous melanoma (PCM) or extracranial metastases (ECM), which was independent of tumor mutational burden. Among MBM, there were fewer computationally inferred immune cell infiltrates, which correlated with lower *TNF* and *IL12B* mRNA levels. Ingenuity pathway analysis (IPA) revealed suppression of inflammatory responses and dendritic cell maturation pathways. MBM also demonstrated a higher frequency of pathogenic *PTEN* mutations and angiogenic signaling. Oxidative phosphorylation (OXPHOS) was enriched in MBM and negatively correlated with NK cell and B cell-associated transcriptomic signatures. Modulating metabolic or angiogenic pathways in MBM may improve responses to immunotherapy in this difficult-to-treat patient subset.

## Introduction

The advent of novel systemic therapies, including immune checkpoint blockade, has raised the 5-year survival for patients with metastatic melanoma from 15% in 2004, up to 30% in the period between 2011 and 2017^1^. However, approximately half of all patients with advanced melanoma will develop brain metastases, leading to death in up to 70% of these patients^2,3^. The management of melanoma brain metastases (MBM) is clinically challenging due to a number of factors, including the heterogeneity of presentation, the presence of neurological symptoms, patterns of intracranial relapse, and the complexity of multi-disciplinary care spanning a combination of surgery, radiotherapy, and systemic therapies^2^. The use of immune checkpoint blockade has demonstrated efficacy in treating some patients with MBM. Indeed, the phase 2 CheckMate 204 trial evaluated the efficacy of combining PD-1 and CTLA-4 inhibition in 94 patients with asymptomatic, non-steroid dependent, untreated MBM and reported an intracranial response rate of 55%, with a partial response rate of 30% and complete response rate of 26%^4^; these response rates are similar to that reported in CheckMate 067, where PD-1 and CTLA-4 combination therapy induced a response rate of 57.6% among patients with extracranial metastases (ECM)^5^. Meanwhile, single-agent PD-1 immunotherapy appears to be less effective in treating MBM, with a response rate (complete or partial) of 26% compared to ECM, where there is a roughly 40% objective response rate^6,7^. While these response rates may not be directly comparable due to differences in prior therapies received among cohorts, there remains much room for improvement in MBM responses to immune checkpoint blockade, which may be aided by a better understanding of the biological differences between MBM and ECM or primary cutaneous melanoma (PCM).

Melanoma is characterized by driver mutations in *BRAF* (47–59%), *NRAS* (22–28%), and *NF1* (14–17%); these mutations have been described in MBM as well, with similar frequencies^8–13^. In addition, *PTEN* alterations (loss-of-function mutations or deletions) are present in 7.3% of primary melanomas and 15.2% of metastatic melanomas and lead to constitutive phosphoinositide 3-kinase (PI3K) pathway activation^14^. The loss of PTEN tumor suppressor activity is important in the early stages of metastasis and is associated with a shorter time to MBM but not ECM^15–17^. Interestingly, higher tumor infiltrating lymphocyte (TIL) density was observed in MBM compared to brain metastases from other cancers^18^, in accordance with the highly immunogenic nature of melanoma that results in greater response rates to immunotherapy^19^. In studies investigating the tumor immune microenvironment of MBM, CD8 + T cells were observed in 90.7% of tumors^20^, and tumors with higher ImmuneScores (a gene expression score representative of greater immune cell infiltrate), were associated with improved overall survival^21^. Despite the contribution of the PI3K pathway to the initiation of MBM and the correlation of PTEN loss with reduced TILs in PCM and local metastases^22^, PTEN protein loss was not found to be related to ImmuneScores in MBM^21^, suggesting other mechanisms at play that remain to be elucidated. Comparison of MBM with patient-matched ECM has revealed that despite a strong presence of immune infiltrate, there are nonetheless significantly lower ImmuneScores and T cell infiltrate in MBM than in ECM^21^. Fischer and colleagues also reported enrichment of oxidative phosphorylation (OXPHOS) and increased immunosuppression in MBM compared to ECM^21^, suggesting that altered metabolism could potentially shape the tumor microenvironment (TME) in MBM. Ultimately, a greater understanding of the TME in MBM is required to optimize therapies for these patients.

In the current study, we sought to compare the transcriptomic and immunologic landscape of MBM relative to PCM and ECM in a large, multi-institutional clinical melanoma cohort, with the goal to consolidate and further clarify recent reports on the immunosuppressive features of MBM. This analysis of a real-world MBM cohort integrating diverse molecular features identified through comprehensive genomic and transcriptomic testing provides advantages over smaller cohorts or clinical trial cohorts that exclude certain subsets of patients. Investigation of the molecular profile and TME unique to MBM can provide further insight into possible treatment vulnerabilities and avenues for future clinical trials.

## Results

### Patient population

In total, 1,314 melanoma samples were available, comprising 94 MBM, 350 PCM, and 870 ECM samples. 48.9% of patients were younger than 67 years old and 61.7% were male, consistent with the established greater incidence of cutaneous melanoma in men than women^23^. ECM comprised tumors from lymph node, lung, liver, and other diverse sites of metastatic disease (Supplementary Table 1). In all samples, DNA was assessed using a 592 gene panel or by whole exome sequencing (WES) and RNA was assessed by whole transcriptome sequencing (WTS) at Caris Life Sciences (Phoenix, AZ).

### Characterization of the immunogenic TME in MBM

**Fig. 1:**
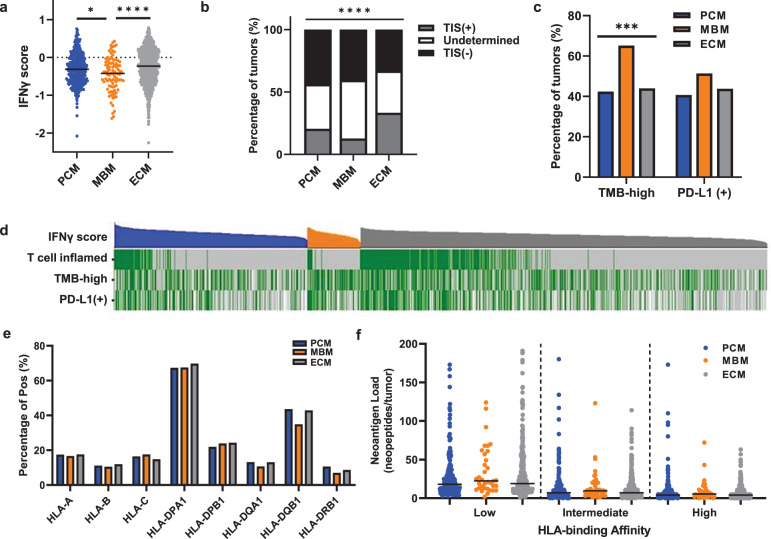
MBM with comparable HLA homozygosity and neoantigen load are less immunogenic than PCM or ECM. **a** IFNγ scores for PCM (blue, *n* = 350), MBM (orange, *n* = 94) and ECM (gray, *n* = 870). Black lines indicate median IFNγ score values. Kruskal–Wallis test with Benjamini-Hochberg correction; ****, corrected *p* < 0.0001; *, corrected *p* < 0.05. **b** PCM, MBM, and ECM were evaluated and classified by T cell-inflamed signature. The percentages of tumors with T cell-inflamed signatures (TIS(+); gray), undetermined (white), and lacking a T cell-inflamed signature (TIS(-); black). Chi-square test; ****p < 0.0001. **c** The percentage of PCM (*n* = 276), MBM (*n* = 72), and ECM (*n* = 681) that are TMB-high and PD-L1(+). Chi-square test; ***, *p* < 0.0005. **d** Oncoplot including IFNγ scores (top, highest to lowest within PCM, blue; MBM, orange; ECM, gray), T cell-inflamed signature (green, T cell-inflamed tumors; gray, others), TMB (high, green; low, gray; white, data not available) and PD-L1 IHC staining (positive, green; negative, gray; white, data not available). **e** Percentage of tumors that are homozygous at HLA-I (HLA-A, HLA-B, and HLA-C) and HLA-II (HLA-DPA1, HLA-DPB1, HLA-DQB1, and HLA-DRB1) loci in PCM, MBM, and ECM. Chi-square test, not significant (*p* > 0.05). **f** Assessment of neoantigen load with low (left), intermediate (middle), and high (right) HLA-binding affinity for PCM (blue), MBM (orange), and ECM (gray). Kruskal–Wallis test; not significant (*p* > 0.05).

To determine whether patients with MBM would likely benefit from immunotherapy, we assessed the immunogenicity of MBM at the transcriptomic level by calculating the interferon-gamma (IFNγ)^24^ score and the T cell-inflamed signature score for each tumor sample in our melanoma cohort^25^. These signatures correlate clinically with response to anti-PD1 therapy independent of tumor mutational burden (TMB)^24^ and immunogenic antigen density^25^. These approaches identified lower IFNγ signature scores (Fig. 1a) and fewer tumors with T cell-inflamed signatures (Fig. 1b) in MBM with respect to PCM and ECM (*p* < 0.05 for IFNγ score and *p* < 0.0001 for percentage of tumors with T cell-inflamed signature). To explore the possibility of lower baseline *IFNG* gene expression in normal brain versus other anatomic sites, we analyzed the GENT2 database of normal and cancer gene expression^26^. Interestingly, *IFNG* gene expression was significantly lower at baseline in normal skin than brain, liver, or lung (Supplementary Fig. 1a). While this data does not consider potential differences in IFNγ scores or IFNγ protein levels, it supports the conclusion that lower IFNγ scores in brain are not simply a reflection of lower baseline levels in normal brain tissue relative to other sites.

Despite these lower IFNγ and T cell-inflamed signature scores, MBM had a higher TMB relative to PCM and ECM (*p* < 0.0001; Fig. 1c), which supports our previous study reporting increased TMB in MBM compared to primary tumors^13^. However, no significant differences were observed in PD-L1 positivity by IHC (Fig. 1c). Oncoplot further showed that tumors with T cell-inflamed signatures clustered together with higher IFNγ scores (Fig. 1d) and significantly correlated in PCM, MBM, and ECM (Supplementary Fig. 1b–d), which has been demonstrated in melanoma and other tumor types. However, neither of these transcriptomic signatures were associated with TMB, indicating that other molecular features may affect immune responses beyond TMB.

We next sought to evaluate the distribution of homozygosity (which can represent parental homozygosity or loss of heterozygosity [LOH]) for human leukocyte antigen (*HLA*)-I and *HLA*-II and neoantigen load, which is associated with TMB status in various cancer types^27,28^. Though TMB and total neoantigen load were positively correlated (Pearson *r* = 0.96, *p* < 0.0001; Supplementary Fig. 2), we failed to detect a significant difference between PCM, MBM, or ECM in the percentage of *HLA* homozygosity at any major loci (Fig. 1e). Likewise, there was no difference in neoantigen load among PCM, MBM, or ECM with different MHC binding affinities (Fig. 1f). This observation agrees with a previous study showing that immunogenic antigen density is not associated with a T cell-inflamed TME in melanoma^25^. Together, we conclude that MBM exhibit a less immunogenic microenvironment identified by both IFNγ score and T cell-inflamed signature, which are independent of the distribution of HLA subtypes, neoantigen load, or TMB.

### Computationally inferred immune cell infiltrates among MBM

**Fig. 2:**
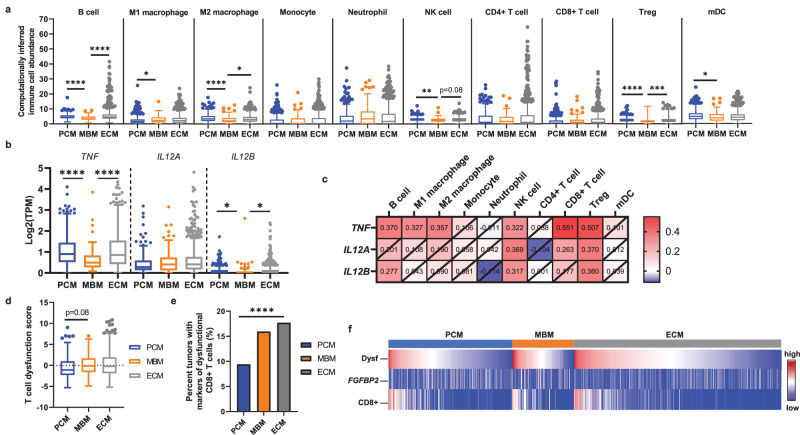
MBM have significantly fewer computationally inferred immune cell infiltrates and more T cell dysfunction. **a** Box plots showing computationally inferred immune cell abundance in PCM, MBM, and ECM using quanTIseq analysis. The data is displayed using the Tukey method for box and whiskers, with the center line indicating the median. Kruskal-Wallis test with Benjamini-Hochberg correction; *, corrected *p* < 0.05; **, corrected *p* < 0.005; ***, corrected *p* < 0.0005; ****, corrected *p* < 0.0001. **b** The mRNA levels of *TNF*, *IL12A*, and *IL12B* were compared between PCM, MBM, and ECM tumor samples. The data is displayed using the Tukey method for box and whiskers, with the center line indicating the median. Kruskal–Wallis test with Benjamini-Hochberg correction; **, corrected *p* < 0.005; ****, corrected *p* < 0.0001 **c** Heat map of Spearman rank correlation coefficients between *TNF*, *IL12A*, and *IL12B* mRNA and immune cell infiltrates calculated from bulk transcriptomic data in MBM. The numbers in each box show the correlation coefficient, where crossed boxes indicate non-significant correlation values (*p* > 0.05). **d** Box plots of T cell exhaustion scores in PCM, MBM, and ECM. The data is displayed using the Tukey method for box and whiskers. Kruskal–Wallis test with Benjamini–Hochberg correction. **e** Percentage of PCM, MBM, and ECM with CD8 + T cells that have high dysfunction scores (composite *z*-score > 1.0) but low *FGFBP2* mRNA levels (less than the median for the whole cohort). *χ*^2^ test; ****p < 0.0001. **f** Oncoplot with T cell dysfunction score (top, highest to lowest within PCM, blue; MBM, orange; ECM, gray), *FGFBP2* mRNA levels (middle), and CD8 + T cell abundance (bottom).

The immune cell populations for PCM, MBM, and ECM were inferred using quanTIseq, a high-throughput computational pipeline specifically designed for RNA-seq data that quantifies the absolute fractions of ten different intratumoral immune cell types^29^. Transcriptomic signatures indicative of B cells, M2 macrophages, natural killer (NK) cells, T regulatory cells (T_regs_) and myeloid dendritic cells (mDC) were significantly reduced in MBM compared to PCM (B cells, *p* < 0.0001; M2, *p* < 0.0001; NK cells, *p* < 0.005; T_regs_, *p* < 0.0001; mDCs, *p* < 0.05) or ECM (B cells, *p* < 0.0001; M2, *p* < 0.05; T_regs_, *p* < 0.005; Fig. 2a). Interestingly, M1 macrophages were significantly higher among MBM relative to PCM (*p* < 0.05).

To better understand cytokine shifts contributing to the altered TME in MBM, we analyzed the gene expression of several critical cytokines between PCM, MBM, and ECM. Interestingly, *TNF* and *IL12B* gene expression in MBM was significantly lower compared to PCM (*TNF*, *p* < 0.0001; *IL12B*, *p* < 0.005) and ECM (*TNF*, *p* < 0.0001; *IL12B*, *p* < 0.005; Fig. 2b), and significantly correlated with computationally inferred B cell, NK cell, and T_reg_ immune cell fractions in MBM (*p* < 0.05; Fig. 2c). Although we observed no difference in CD8+ immune cell fraction measured by quanTIseq between PCM, MBM, and ECM, *TNF* also correlated with CD8 + T cells in MBM tumors (Fig. 2c). Together, these results suggest that immune cell infiltration is overall reduced in MBM especially compared to PCM, and associated reductions in cytokine levels in MBM may contribute to the more immune excluded phenotype.

### T cell dysfunction and STING scores among MBM

Within the heterogeneous TME, dysfunctional CD8 + T cells may be observed, as characterized by increased expression of inhibitory checkpoint molecules such as TIM3, LAG3, and PD-1, as well as the loss of classical CD8 + T cell effector functions including proliferative capacity and cytotoxicity^30–32^. Therefore, we were interested in further defining the state of the CD8 + T cells infiltrated in MBM, even though the transcriptomic data suggested that the total abundance of CD8 + T cells was comparable between PCM, MBM, and ECM (Fig. 2a). We evaluated the T cell dysfunction scores—defined by the sum of z-scores of *HAVCR2* (TIM3), *LAG3*, and *PDCD1* (PD-1) mRNA levels—as well as mRNA levels for the cytotoxic T cell marker *FGFBP2*^30^. T cell dysfunction scores trended toward higher in MBM relative to PCM (*p* = 0.08; Fig. 2d), and among tumors with non-zero CD8 + T cell infiltration, the percentage of tumors with transcriptomic signatures indicating non-functional CD8 + T cells was significantly lower among MBM (*p* < 0.0001; non-functional CD8 + T cells were defined by high dysfunction scores [composite z-score >1.0] but low *FGFBP2* mRNA levels [less than median TPM for cohort]; Fig. 2e). These results contribute toward a picture of MBM as possessing a more immune-suppressed TME, particularly relative to PCM. Tumors with transcriptomes associated with high CD8 + T cell infiltration also exhibited greater dysfunction scores regardless of anatomic site (i.e., PCM, ECM, MBM; Fig. 2f).

T cell responses have also been shown to be affected by STING signaling^33,34^, with various predicted outcomes on immune response and metastasis^35–37^. A positive association was observed between composite STING scores (comprising mRNA levels of key regulators of the STING pathway [*TMEM173*, *CGAS*, *CCL5*, *CXCL10* and *IRF3*]), computationally inferred CD8 + T cell infiltration, and T cell dysfunction scores among all melanoma tumors (*p* < 0.0001; Supplementary Fig. 3a–c) and among each anatomic site (*p* < 0.0001; Supplementary Fig. 3d). Despite this correlation, T cell dysfunction scores trended toward higher among MBM compared to PCM (Fig. 2d), while STING (*TMEM173*) mRNA levels and STING scores were significantly reduced in MBM compared to both PCM and ECM (*p* < 0.05; Supplementary Fig. 3e, f). These data suggest that further analysis of the effect of STING signaling on tumor immunity in MBM may be warranted.

### PTEN mutation-associated angiogenic factors among MBM

**Fig. 3:**
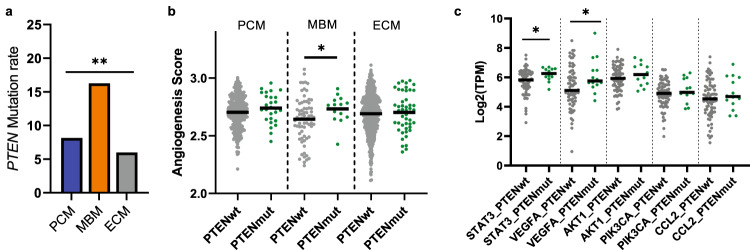
Association of angiogenic factors with *PTEN* mutations in MBM. **a** The percentage of PCM (blue, *n* = 332), MBM (orange, *n* = 86), and ECM (gray, *n* = 801) with *PTEN* mutations. Chi-square test; **p < 0.005. **b** ssGSEA analysis for normalized enrichment scores (NES) of hallmark angiogenesis pathway comparing tumors with *PTEN* mutations (green) and without *PTEN* mutations (gray) within PCM, MBM, and ECM groups. Black line indicates median. Mann-Whitney test; *p < 0.05. **c** The mRNA levels of key regulatory genes (*STAT3*, *VEGFA*, *AKT1*, *PIK3CA*, and *CCL2*) in the angiogenesis pathway were cross compared between MBM tumors with *PTEN* mutations (green) and without *PTEN* mutations (gray). Black line indicates median. Mann-Whitney test; *p < 0.05.

To determine the frequency of non-synonymous somatic mutations enriched in MBM that could possibly contribute to reduced immune responses or altered immune cell trafficking, DNA sequencing data was analyzed. *BRAF*, *NRAS*, and *NF1* are the most commonly mutated genes in cutaneous melanomas^38^, while *PTEN* loss is associated with brain metastasis^15^; therefore, we examined mutations in these key genes among PCM, MBM, and ECM. Among MBM samples with available NGS data, there were 44/92 samples with pathogenic mutations affecting *BRAF* (47.8%), 20/91 with *NRAS* (22.0%), and 28/41 with *NF1* (68.3%; Supplementary Data 2). When assessing for molecular or immunologic correlates by driver mutation status, TMB-high tumors were more prevalent in *BRAF*-mut MBM relative to *BRAF*-mut PCM, and neutrophils were enriched in *NRAS*-mut MBM relative to *NRAS*-mut PCM (Supplementary Fig. 4). Driver mutations notwithstanding, our data demonstrate significantly higher pathogenic *PTEN* mutation rates in MBM (16.3%) with respect to PCM (8.1%) and ECM (6.0%, *p* < 0.005; Fig. 3a and Supplementary Data 2). Previous studies found that *PTEN*-deficient tumors have higher levels of *VEGFA* and *STAT3*, which promote angiogenesis and impair the trafficking of immune cells into the TME^22,39^. In addition, inhibition of the JAK-STAT3 pathway in *PTEN*-null prostate cancers increased immune infiltration and promoted a more immunogenic TME^40^. Therefore, we sought to evaluate the correlation of pathogenic (loss-of-function) *PTEN* mutations with angiogenesis via single sample gene set enrichment analysis (ssGSEA) in PCM, MBM, and ECM. A significant enrichment of angiogenic signaling was observed in *PTEN*-mut compared to *PTEN* wild-type (wt) MBM (*p* < 0.05), but not in PCM or ECM (Fig. 3b). Moreover, simultaneous elevation of *VEGFA* (*p* < 0.05) and *STAT3* (*p* < 0.05) mRNA levels was observed in *PTEN*-mut MBM (Fig. 3c), suggesting a relationship between *PTEN*-mut and angiogenic signaling uniquely in MBM, which may contribute to its altered TME.

### Differentially expressed genes and associated pathways in MBM

**Fig. 4:**
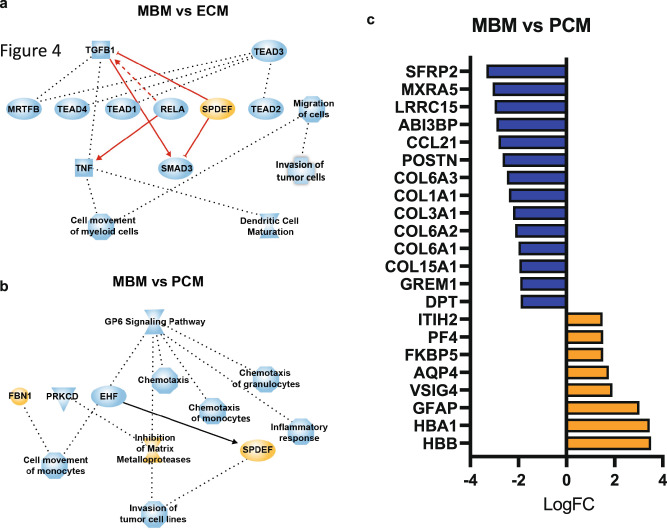
Differentially expressed genes in MBM compared to PCM and ECM. Ingenuity pathway analysis (IPA) shows a significant enrichment of pathways in MBM compared to ECM (**a**) and PCM (**b**). Orange node, enrichment in MBM; blue node, enrichment in ECM or PCM. **c** Differentially expressed genes (Log2FC; adjusted *p* < 0.01) in MBM compared to PCM. Only genes that were differentially expressed in MBM compared to both PCM and ECM are shown. Orange bars indicate genes that were upregulated in MBM and blue bars indicate genes that were downregulated in MBM.

Transcriptomic profiles were assessed to determine if they would explain the differences in inferred immune cell abundance between MBM and PCM or ECM. Significantly differentially expressed genes (DEGs) were identified between MBM and ECM and between MBM and PCM (Supplementary Data 3), followed by the assessment of differentially regulated pathways via Ingenuity Pathway Analysis (IPA). Movement of myeloid cells and DC maturation pathways were less enriched in MBM compared to ECM, and chemotaxis and inflammatory responses were less enriched in MBM compared to PCM (Fig. 4a). In addition, signaling involving the collagen receptor platelet glycoprotein VI (GP6) was less enriched in MBM compared to PCM (Fig. 4b), which likely represents the differing milieu of the brain relative to collagen-rich skin.

To better understand the unique transcriptomic features of MBM, we further examined DEGs between MBM and PCM/ECM (Supplementary Data 3). Hemoglobin subunit beta (*HBB*) and hemoglobin subunit alpha 1 (*HBA1*) mRNA levels were significantly upregulated in MBM (Fig. 4c), although their upregulation in MBM may reflect a hemorrhagic component of the tumor as opposed to a true biological difference. Furthermore, v-set and immunoglobulin domain containing 4 (*VSIG4*) and platelet factor 4 (*PF4*), which are negatively associated with T cell function and proliferation^41–43^, were overexpressed in MBM relative to PCM (Fig. 4c). Conversely, extracellular matrix-related genes, including several collagens, were downregulated in MBM relative to PCM, as expected due to anatomic site (Fig. 4c). Notably, *CCL21*, which stimulates chemotaxis of mDCs and helps leukocytes traverse the blood-brain barrier^44^, was downregulated among MBM compared to PCM, illustrating potential cytokine shifts supporting altered immune infiltration in MBM. While there was no correlation of mDC abundance measured by quanTIseq with *CCL21* mRNA, there was a moderate positive correlation between *CCL21* mRNA and CD8 + T cell abundance in MBM (Spearman *r* = 0.32, *p* = 0.0017; Supplementary Fig. 5a, b). These observations suggest a distinct environment in MBM that might impact on both the abundance and functionality of infiltrating immune cells.

### Enrichment of oxidative phosphorylation pathway in MBM

**Fig. 5:**
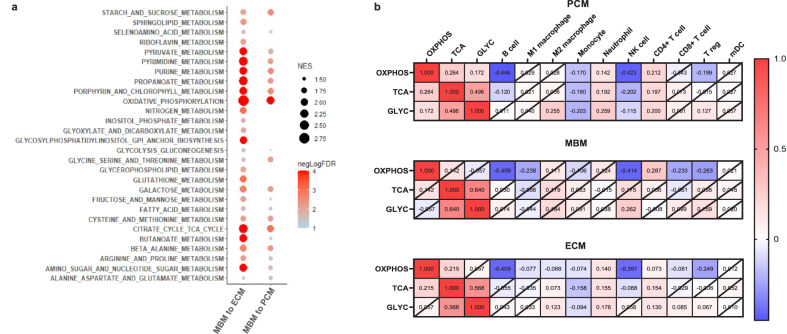
MBM show enrichment in oxidative phosphorylation, TCA cycle, and glycolysis gluconeogenesis pathways. **a** GSEA analysis demonstrates a significant enrichment in of KEGG metabolic pathways in MBM versus PCM (left lane) or ECM (right lane). The normalized enrichment score (NES) is indicated by the size of the dot and the -log10 *p* value (cut off FDR < 0.05) is indicated by the color of the dot. **b** Heat maps of Spearman rank correlations between NES of OXPHOS, TCA cycle (TCA), and glycolysis (GLYC) determined from ssGSEA and computationally inferred immune cell abundance in PCM, MBM, and ECM. The numbers in each box show the correlation coefficient, where crossed boxes indicate non-significant correlation values (*p* > 0.05).

Fischer et al. and Biermann et al. reported significant immunosuppression and enrichment of oxidative phosphorylation (OXPHOS) in MBM compared to ECM^21,45^. To confirm these findings in our large melanoma cohort, we performed GSEA analysis for KEGG metabolic pathways. This analysis demonstrated that the most significant pathways enriched in MBM were OXPHOS, the TCA cycle, various amino acid metabolism, followed by glycolysis (Fig. 5a and Supplementary Data 4), which was particularly striking when comparing MBM to ECM. Interestingly, individual normalized enrichment scores (NES) for OXPHOS and the TCA cycle were not correlated in MBM and only weakly in PCM and ECM (Fig. 5b). In agreement with the lack of correlation between OXPHOS and the TCA cycle in MBM, we noted that OXPHOS, but not TCA cycle or glycolysis enrichment, significantly correlated with transcriptional signatures indicating a lower abundance of B cells (Spearman *r* = −0.41), M1 macrophages (Spearman *r* = −0.238), NK cells (Spearman *r* = −0.41), CD8 + T cells (Spearman *r* = −0.23), and T_regs_ (Spearman *r* = −0.26, *p* < 0.05 for all), demonstrating a potential negative impact of OXPHOS on these immune infiltrates in a TCA-independent manner (Fig. 5b).

## Discussion

The management of MBM remains an area of complexity and clinical challenge for healthcare providers treating patients with advanced melanoma. While the promise of immune checkpoint blockade in treating MBM has been an important breakthrough, there are still many unanswered questions, including how to select patients who will benefit from immunotherapy and how to overcome resistance. As such, an improved understanding of the unique biological features of MBM and the brain microenvironment is critical to identifying additional barriers and opportunities for therapy. In this study, we utilized a large cohort of 94 MBM, 350 PCM, and 870 ECM samples for which comprehensive genomic and transcriptomic profiling was readily available, in order to assess underlying biological differences in MBM compared to PCM and ECM. To our knowledge, this is one of the largest such studies to date.

Given the molecular heterogeneity of brain metastases, the role of putative biomarkers such as PD-L1 and TMB may have limited predictive value for MBM. Prior studies have shown that PD-L1 expression may vary between anatomic sites and does not clearly correlate with the presence of CD8 + T cells and other immune infiltrates^13,20,46^. Meanwhile, TMB is related to neoantigen load and correlates with improved outcomes in several cancer types, but the absolute relationship between TMB and immunogenicity or response to immunotherapy may also depend upon the tumor type or the specific genomic landscape and immune repertoire of each patient^47^. For instance, Biermann and colleagues demonstrated spatial heterogeneity of type-I IFN responses in MBM despite broad expression of antigen presentation genes^45^. The current study further corroborates that PD-L1, TMB status, or neoantigen load are not necessarily representative of functional immunogenicity, and may be poor biomarkers of immunotherapy response in MBM.

While a healthy blood-brain barrier normally restricts T cell extravasation into the brain, the high concentration of tumor-associated T cells in MBM illustrates a breakdown of this barrier^18,48^ and necessitates a thorough understanding of factors affecting the quantity and function of MBM immune infiltrate. Despite a robust T cell infiltrate in MBM, studies have found reduced ImmuneScores, mDCs, NK/T cells, B lineage cells, and neutrophils and increased exhausted T cells in matched and unmatched MBM cohorts relative to PCM or ECM^21,45,49^. Alvarez-Breckenridge et al. furthermore found that while absolute T cell infiltration did not predict response to immune checkpoint blockade in MBM, peripheral T cell clonal expansion was associated with improved responses, and intracranial clonally expanded T cells were more likely to be exhausted^50^. Our analysis is consistent with the above findings, identifying reduced immune cell recruitment, lower IFNγ and T cell-inflamed signature scores, and transcriptional signatures suggesting a trend toward increased dysfunctional CD8 + T cells in MBM. The decrease in computationally inferred B cells in MBM was the most pronounced and significant immune cell difference relative to both PCM and EMC, meriting further investigation as humoral immunity is increasingly recognized to play a significant role in promoting an inflammatory milieu and helping mediate responses to immune checkpoint blockade in melanoma^51,52^. These data are a timely addition to single-cell analyses of MBM showing the full range of intratumoral differentiation from naïve B cells to activated B cells to plasma cells, with increased plasma cell aggregates present in MBM compared to ECM^45,49^.

While multiple mechanisms may contribute to the distinct TME of MBM, our correlative analysis suggests that cytokine shifts, genomic differences in rates of pathogenic *PTEN* mutations, and altered metabolism may each contribute to these observations. Interestingly, Fischer and colleagues found no differences in PTEN protein loss that could explain the reduced immunogenicity they observed in MBM^21^. Notwithstanding, their group previously reported that loss of PTEN upregulated *VEGF* and *CCL2* and resulted in lower responses to immunotherapy in a mouse model of melanoma and that VEGF and PTEN protein expression inversely correlated in clinical samples^22^. Together with our findings that pathogenic *PTEN* mutations correlate with angiogenic signaling in MBM, these results emphasize the potential role of unrestricted PI3K-AKT signaling in dampening immune responses. Therefore, we hypothesize that patients exhibiting loss of PTEN tumor suppressor activity may be more likely to benefit from a combined therapeutic approach involving anti-angiogenesis therapy with immunotherapy, as is currently being investigated in a phase 2 clinical trial enrolling untreated MBM patients (NCT03175432).

The most striking finding from GSEA was the enrichment of OXPHOS metabolic pathways in MBM, agreeing with major findings by Fischer et al. and Biermann et al. in smaller cohorts^21,45^. Interestingly, OXPHOS may be a mechanism of metastatic spread^53^ and resistance to MAPK inhibitors in melanoma^54,55^. IACS-010759, an OXPHOS inhibitor currently in early-phase trials, inhibited MBM formation and prolonged survival in a spontaneous MBM mouse model^21^. Our data suggest that the metabolic diversity enacted through OXPHOS and glycolysis fluctuation present in a large percentage of melanomas^56^ may also occur in MBM, which displayed significant enrichment of OXPHOS and the TCA cycle relative to PCM and ECM, and to a lesser degree glycolysis and gluconeogenesis relative to ECM tumors. This conclusion is further supported by findings by Biermann et al. indicating heterogeneity of OXPHOS metabolism in MBM at both a single cell level and spatially^45^. Beyond metabolic flexibility, the emerging field of immunometabolism suggests there may be a connection between metabolism and immune cell effector functions^57^, potentially explaining the negative correlation between OXPHOS and NK cell, M1 macrophage, B cell, CD8 + T cell, and T_reg_ abundance determined from bulk transcriptomic data in MBM. In agreement with these findings, Li et al. determined that a subset of CD8 + T cells with high OXPHOS promotes immunotherapy resistance in melanoma^58^. Taken together, our findings highlight several possible immunosuppressive mechanisms in MBM that underscore the need for a deeper understanding of potential therapeutic vulnerabilities and biomarkers of immunotherapy response in MBM.

Considering that multiple immune inhibitory mechanisms may be simultaneously active within MBM, a number of clinical trials are investigating whether combinations of BRAF, MEK1/2, AKT/PI3K, CTLA-4, PD-1, and VEGF targeted therapy will be more successful, as opposed to monotherapy approaches^2,59,60^. Investigation of mechanisms contributing to dampened immune responses in MBM, as we present here, is essential to improve long-term responses to these therapies and expand the number of eligible patients. To this point, two phase I/II trials are enrolling patients to evaluate the combination of metformin (an inhibitor of OXPHOS) with vemurafenib (NCT01638676) or dabrafenib and trametinib (NCT02143050) in patients with metastatic melanoma with activating mutations in the *BRAF* gene. However, additional pharmacologic approaches for modulating OXPHOS and tumor immunogenicity in MBM should also be explored.

One major limitation of the present study is that this retrospective, correlative analysis does not allow the precise determination of mechanisms connecting cytokine signaling, *PTEN*-mut/angiogenesis, and OXPHOS to reduced immunogenicity in MBM; the bulk-profiling nature of the study also further limits our understanding of intra-lesional heterogeneity. Likewise, while we only detected a trend toward increased T cell dysfunction scores in MBM relative to PCM, examination of alternative transcriptional signatures representing T cell exhaustion or dysfunction could be informative. Future experimental studies can utilize the hypothesis-generating results from this large, “real-world” MBM cohort to decipher mechanisms involved in regulating the TME in MBM. An additional limitation is the lack of clinical annotation for this melanoma cohort, including stage and detailed treatment information (i.e., whether patients had received therapy prior to tissue collection), as systemic targeted therapy or immunotherapy is known to affect the tumor microenvironment of MBM^49^. Finally, the ECM and MBM cohorts were not strictly exclusive of non-cutaneous primary sites; however, the rarity of such tumors likely limits any potential effect on results due to our relatively large cohort size. Despite these limitations, the overall concordance of our results with the two prior studies by Fischer et al. and Biermann et al., each of which used smaller sample sizes and different analytic techniques^21,45^, substantially supports our conclusions regarding immunosuppression in MBM.

In summary, this retrospective study of 1314 melanoma samples contributes to existing data that TMB, PD-L1, and other traditional biomarkers are poor predictors of immunogenicity in MBM, which instead may engage multiple intersecting pathways for immune evasion, including cytokine signaling, angiogenesis, and OXPHOS metabolism. Future studies must define how targeting OXPHOS affects the tumor immune microenvironment mechanistically and determine the clinical benefit of targeting such pathways to improve immunotherapy responses in MBM.

## Methods

### Patient samples

A multi-institutional cohort of 1,314 patient samples, including PCM (*n* = 350), MBM (*n* = 94), and ECM (*n* = 870) from the Caris Life Sciences database were analyzed in a CLIA/CAP-certified laboratory (Caris Life Sciences, Phoenix, AZ). Analyses performed include next-generation sequencing (NGS), whole transcriptome sequencing (WTS), and immunohistochemistry (IHC) for molecular and genomic features including tumor mutational burden, tumor mutations, HLA homozygosity, neoantigen load, PD-L1 positivity, and mRNA levels. A breakdown of patient specimens, including the number of samples, tumor site, age, and sex, is shown in Supplementary Table 1. The data was not further analyzed according to age or sex.

### Next-generation sequencing

NGS was performed on genomic DNA isolated from formalin-fixed paraffin-embedded (FFPE) tumor samples using the NextSeq or NovaSeq 6000 platforms (Illumina, Inc., San Diego, CA). Prior to molecular testing, tumor enrichment was achieved by harvesting targeted tissue using manual microdissection techniques. For NextSeq-sequenced tumors, a custom-designed SureSelect XT assay was used to enrich 592 whole-gene targets (Agilent Technologies, Santa Clara, CA). For NovaSeq whole exome-sequenced tumors, a hybrid pull-down panel of baits designed to enrich for more than 700 clinically relevant genes at high coverage and high read-depth was used, along with another panel designed to enrich for additional >20,000 genes at a lower depth. A 500 Mb SNP backbone panel (Agilent Technologies, Santa Clara, CA) was added to assist with gene amplification/deletion measurements and other analyses. All variants were detected with >99% confidence, with an average sequencing depth of coverage of > 500 and an analytic sensitivity of 5%. This test has a sensitivity to detect as low as approximately 10% population of cells containing a mutation in all exons from the high read-depth clinical genes and 99% of all exons in the 20 K whole exome regions. Genetic variants identified were interpreted by board-certified molecular geneticists and categorized as ‘pathogenic,’ ‘likely pathogenic,’ ‘variant of unknown significance,’ ‘likely benign,’ or ‘benign,’ according to the American College of Medical Genetics and Genomics (ACMG) standards. When assessing mutation frequencies of individual genes, ’pathogenic,’ and ‘likely pathogenic’ were counted as mutations while ‘benign’, ‘likely benign’ variants, and ‘variants of unknown significance’ were excluded. Variants detected were mapped to reference genome (hg19), and well-established bioinformatics tools such as BWA, SamTools, GATK, and snpFF were incorporated to perform variant calling functions; germline variants were filtered with various germline databases, including 1000 Genomes and dbSNP.

### Tumor mutational burden

Tumor mutational burden (TMB) was measured (592 genes and 1.4 megabases [MB] sequenced per tumor) by counting all non-synonymous missense mutations found per tumor that had not been previously described as germline alterations according to dbSNP and 1KG databases. A cutoff of ≥10 mutations per MB (mt/MB) was used based on the KEYNOTE-158 pembrolizumab trial, which showed that patients with a TMB of ≥ 10 mt/MB across several tumor types had higher response rates than patients with a TMB of <10 mt/MB^61^.

### Neoantigen load

HLA genotyping and immune epitope prediction of peptides was performed to quantify neoantigen load. HLA genotyping was performed using arcasHLA^62^. If a single HLA genotype was detected, the specimen was classified as “homozygous”, which can occur due to parental homozygosity or HLA loss of heterozygosity (LOH). MHC-I binding prediction was performed for all HLA allele/peptide combinations for each tumor using the NetMHCpan v4.0 method in the Immune Epitope Database (IEDB) online resource at http://tools.iedb.org/mhci. Peptide affinity for MHC-I molecules was categorized based on guidelines described on the IEDB website: high affinity [inhibitory concentration 50% (IC50) < 50 nmol/L], intermediate affinity (IC50 > 50 nmol/L and < 500 nmol/L), low affinity (IC50 > 500 and < 5000 nmol/L), and no affinity (IC50 > 5000 nmol/L).

### Whole transcriptome sequencing

Tumor-specific RNA was extracted from microdissected FFPE specimens with at least 10% tumor content using a Qiagen RNeasy FFPE Kit. RNA quality and quantity were determined using the Agilent TapeStation. Biotinylated RNA baits were hybridized to the synthesized and purified cDNA targets, and the bait-target complexes were amplified in a post-capture PCR reaction. WTS was performed using the Illumina Novaseq 6500 to an average of 60 M reads. Raw data were demultiplexed by Illumina Dragen BioIT accelerator, trimmed, counted, PCR-duplicates removed, and aligned to the human reference genome hg19 by STAR aligner. Transcripts per million (TPM) were calculated using the Salmon expression pipeline. Differentially expressed genes (DEGs) were determined by calculating log2 fold-change between defined groups, and genes were considered significantly different when adjusted *p* < 0.05. Ingenuity Pathway Analysis (IPA) was used to determine differentially regulated pathways between PCM and MBM and between MBM and ECM. Significantly enriched pathways in MBM relative to PCM or ECM were determined using Gene Set Enrichment Analysis (GSEA) to calculate normalized enrichment score (NES) and significance level adjusted for multiple hypothesis testing^63^. Single sample GSEA (ssGSEA) was used to calculate individual enrichment scores for samples relative to specific gene sets (e.g., angiogenesis score).

### Immune cell abundance and scoring

Immune cell abundance was calculated from WTS data using quanTIseq^29^. QuanTIseq is a computational pipeline that measures the abundance of ten immune cell populations from bulk transcriptomic data. The algorithm is highly validated against flow cytometry and immunohistochemistry for the deconvolution of absolute fractions of myeloid dendritic cells (DCs), T regulatory (T_regs_), CD8+ and CD4 + T cells, natural killer (NK) cells, neutrophils, monocytes, M1 and M2 macrophages, and B cells. Signature scores were calculated using defined gene sets for interferon (IFN)^24^, T cell-inflammation^25^, exhausted T cells (*HAVCR2*, *LAG3*, *PDCD1*), and STING pathway (*TMEM173*, *CGAS*, *CCL5*, *CXCL10*, *IRF3*)^64,65^.

### Immunohistochemistry

Immunohistochemistry (IHC) was performed on FFPE sections on glass slides. Automated staining techniques were utilized per the manufacturer’s instructions and were optimized and validated per CLIA/CAP and ISO requirements. The primary antibody used for PD-L1 was clone SP142 (Roche/Ventana Medical Systems Inc., Oro Valley, AZ, cat. #740-4859), which is provided by the manufacturer at the optimal dilution for use. A board-certified pathologist evaluated all IHC results independently, and the staining was regarded as positive if its intensity on the membrane of the tumor cells was ≥ 2+ (on a semiquantitative scale of 0–3: 0 for no staining, 1+ for weak staining, 2+ for moderate staining, or 3 + for strong staining) and the percentage of positively stained cells was ≥ 5%.

### Statistics

Kruskal-Wallis test with Benjamini-Hochberg post-hoc correction or two-sided Mann-Whitney test were used to determine significant differences in continuous data between groups analyzed. Fisher exact or Chi-square tests were used to determine significant differences in the prevalence of binary variables between groups. Spearman rank or Pearson correlation were used for correlation analyses. All measurements were taken from distinct samples. Standard statistical methods in R (version 4) and GraphPad Prism (version 9) were employed to analyze the data. For all tests, *p* < 0.05 (or corrected *p* < 0.05, where applicable) was considered statistically significant.

### Ethics statement

This study was conducted in accordance with guidelines set forth in the Declaration of Helsinki, Belmont report, and U.S. Common rule. Per 45 CFR 46.101(b)(4) for retrospective studies using de-identified biospecimens and clinical data, this study was considered IRB exempt by Western IRB and informed consent was not required.

### Reporting summary

Further information on research design is available in the Nature Research Reporting Summary linked to this article.

### Supplementary information

Supplementary Material Merged

Summarized molecular data for cohort

Differentially expressed genes between MBM and ECM or PCM

GSEA comparing MBM to ECM or PCM

REPORTING SUMMARY

## Data Availability

The datasets generated and/or analyzed during the current study are available for replication and verification purposes from the corresponding author on reasonable request. The de-identified DNA and RNA sequencing data are owned by Caris Life Sciences and cannot be publicly shared due to the data usage agreement signed by Dr. Heinz-Josef Lenz at Keck School of Medicine of USC. Qualified researchers can apply for access to these data by contacting Joanne Xiu, PhD at jxiu@carisls.com, submitting a brief proposal, and signing a data usage agreement. The summarized sequencing data are available within the Supplementary Materials for this paper and at https://figshare.com/s/4f0dbb3aaf58d17f4615.
