# Supplementary material for: Multi-omic profiling reveals discrepant immunogenic properties and a unique tumor microenvironment among melanoma brain metastases
Source: NPJ Precis Oncol. 2023 Nov 14;7:120. doi: 10.1038/s41698-023-00471-z (PMC10646102; doi:10.1038/s41698-023-00471-z)
Supplement: Supplementary file 1 — Supplementary Material Merged [file 41698_2023_471_MOESM1_ESM.pdf]

Supplementary Table 1: Study cohort							
		Age, N (%)			Sex, N (%)		
Site	N	<67	≥67	*p* value	Male	Female	*p* value
PCM	350	167 (47.71)	183 (52.29)	NS	200 (57.14)	150 (42.86)	**
ECM-skin	123	50 (40.65)	73 (59.35)	**	80 (65.04)	43 (34.96)	**
ECM-lymph node	241	138 (57.26)	103 (42.74)	**	165 (68.46)	76 (31.54)	**
ECM-lung	105	45 (42.86)	60 (57.14)	NS	73 (69.52)	32 (30.48)	**
ECM-liver	48	18 (37.50)	30 (62.50)	NS	38 (79.17)	10 (20.83)	**
ECM-other	353	181 (51.27)	172 (48.73)	NS	185 (74.47)	168 (25.53)	NS
MBM	94	43 (45.74)	51 (54.26)	NS	70 (74.47)	24 (25.53)	**

Supplementary Table 1. Study cohort. Numbers of tumor samples (N) are shown for PCM, MBM, and ECM that were divided into skin, lymph node, lung, liver, and other based on the site of metastasis. Fisher's exact test; **, $p < 0.01$. PCM, primary cutaneous melanoma; ECM, extracranial metastasis; MBM, melanoma brain metastasis; NS, not significant.

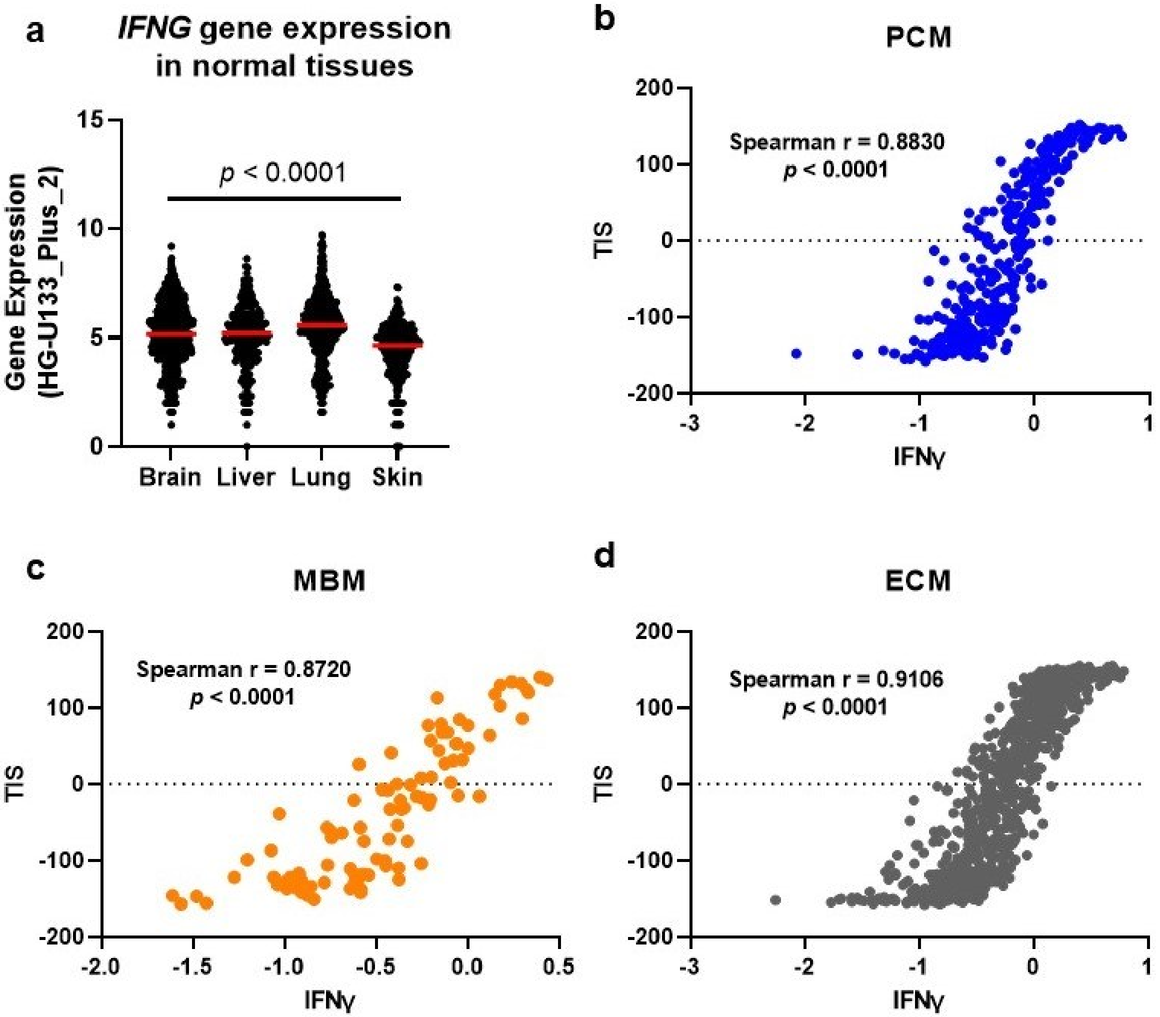

Supplementary Figure 1. Analysis of IFNγ and T cell inflammation scores. a, Expression data (HG-U133_Plus_2) was downloaded from the GENT2 database for *IFNG* in normal brain, liver, lung, and skin. Red line indicates the median. *p*-value was determined using non-parametric Kruskal-Wallis test. **b-d,** Spearman rank correlation of IFNγ scores and T cell inflammation scores (TIS) in PCM (**b**), MBM (**c**), and ECM (**d**).

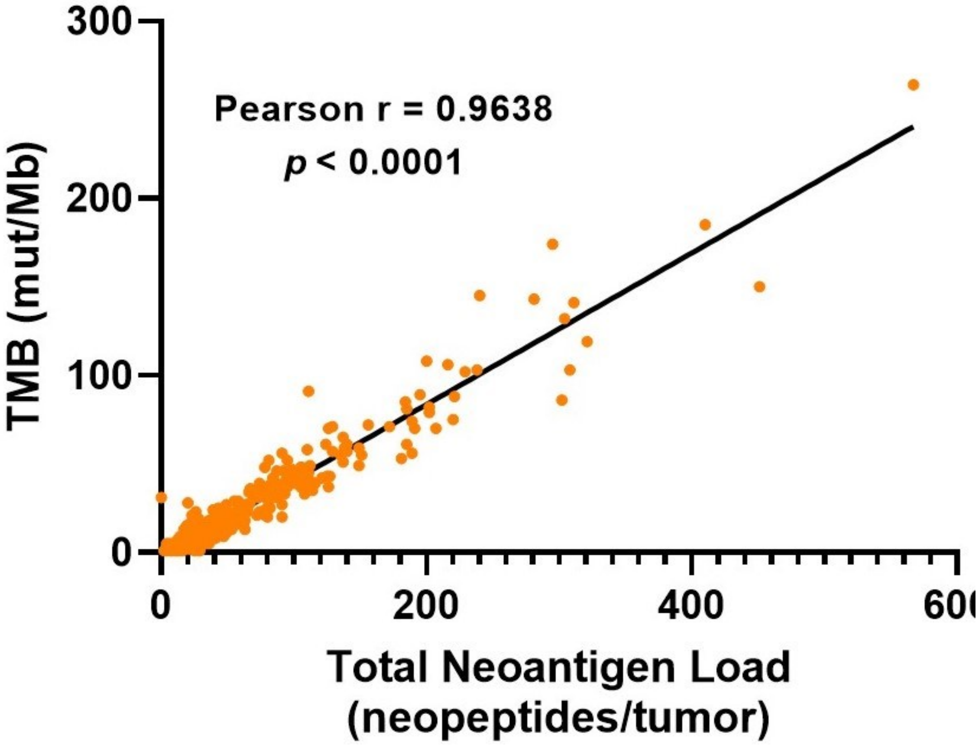

Supplementary Figure 2. Correlation between TMB and total neoantigen load. Pearson correlation between TMB (mut/Mb) and neoantigen load (neopeptides/tumor) in all melanoma samples with matched TMB and neoantigen load data (n=451).

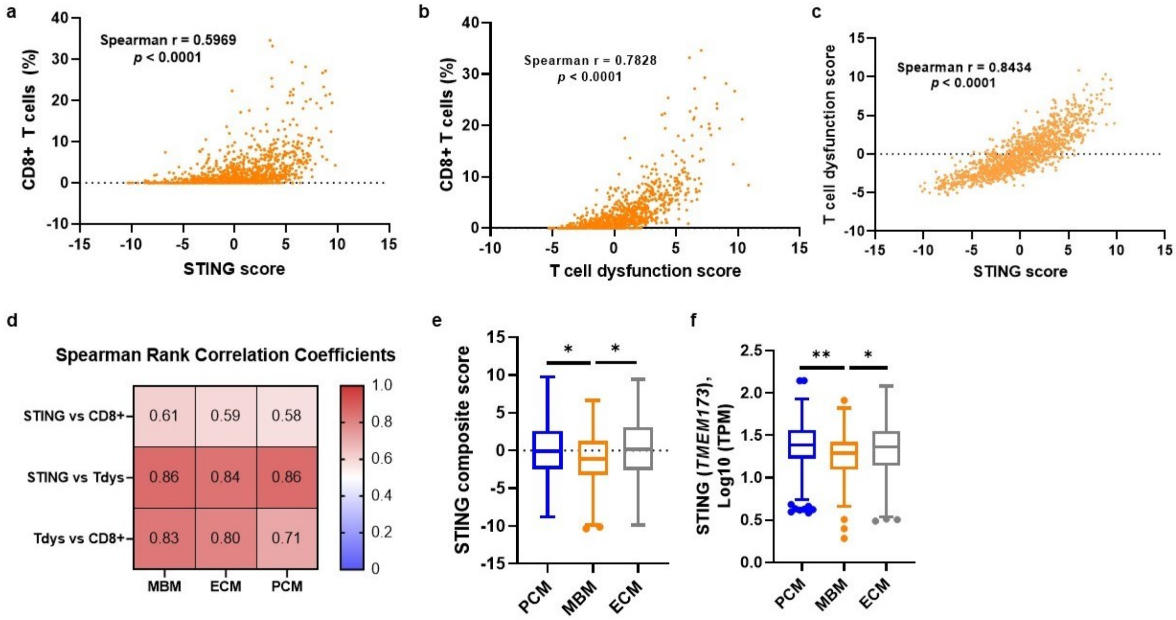

Supplementary Figure 3. MBM display reduced STING pathway gene expression. a, Spearman rank correlation of STING composite score and CD8+ T cell fraction in entire melanoma cohort. **b,** Spearman rank correlation of computationally inferred CD8+ T cell fraction and T cell dysfunction scores in entire melanoma cohort. **c,** Spearman rank correlation of T cell dysfunction scores and STING scores in entire melanoma cohort. **d,** Heat map of Spearman rank correlation coefficients for STING scores, CD8+ T cells, and T cell dysfunction scores (Tdys). $p < 0.0001$ for all correlations. **e**, Box plot of composite STING score for PCM (blue), MBM (orange), and ECM (grey). The data is displayed using the Tukey method for box and whiskers, with the center line indicating the median. Kruskal-Wallis test with Benjamini-Hochberg correction. **f,** Box plot of *TMEM173* (STING) mRNA levels for PCM (blue), MBM (orange), and ECM (grey). The data is displayed using the Tukey method for box and whiskers, with the center line indicating the median. Kruskal-Wallis test with Benjamini-Hochberg correction. For **e** and **f**, *, corrected $p < 0.05$; **, corrected $p < 0.005$.

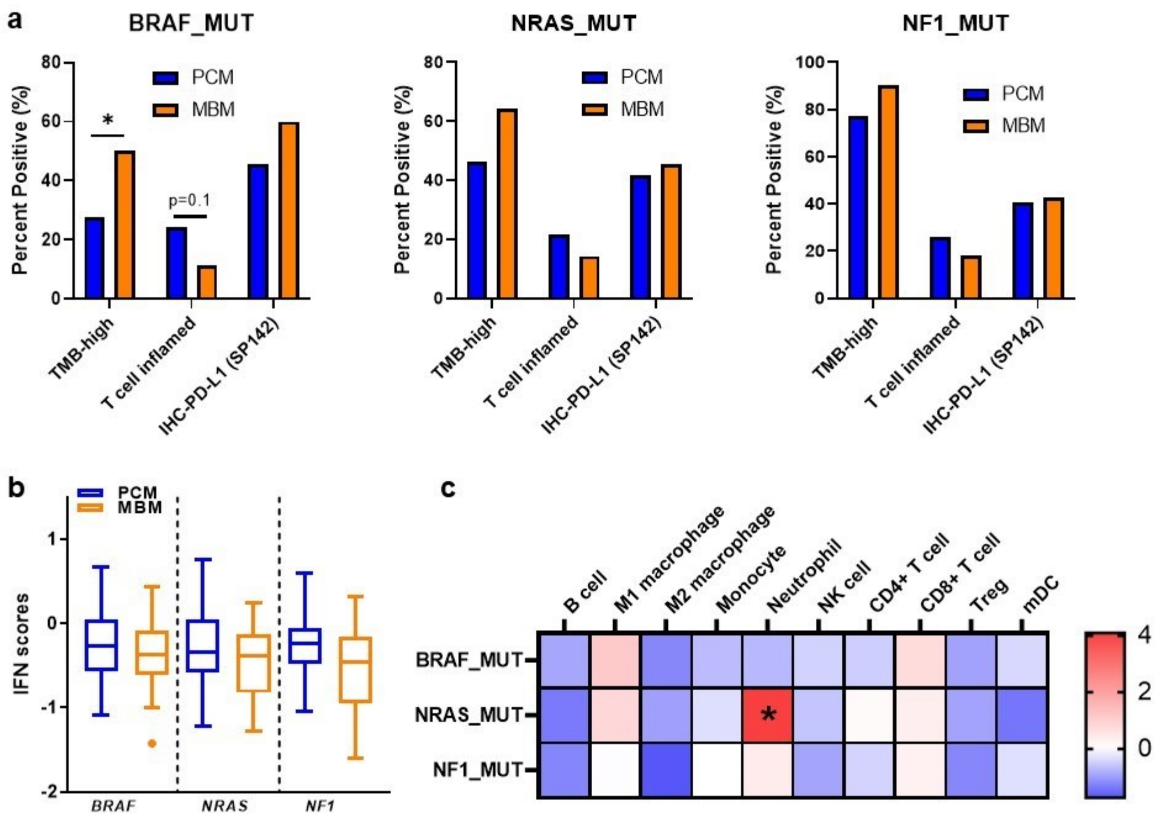

Supplementary Figure 4. Relationship of *BRAF*, *NRAS*, and *NF1* mutations with tumor immune profile. a, Percentage of tumors positive for TMB-high status, T cell-inflamed tumor score, and PD-L1 (by IHC) in PCM and MBM tumors with *BRAF*, *NRAS*, or *NF1* mutations. Mann-Whitney test; *, $p < 0.05$. **b,** Comparison of IFN scores between PCM and MBM with *BRAF*, *NRAS*, or *NF1* mutations. The data is displayed using the Tukey method for box and whiskers. **c,** Heat map of fold-change computationally inferred immune cell abundance in MBM relative to PCM with *BRAF*, *NRAS*, or *NF1* mutations. Red, increased in MBM; blue, decreased in MBM. Mann-Whitney test; *, $p < 0.05$.

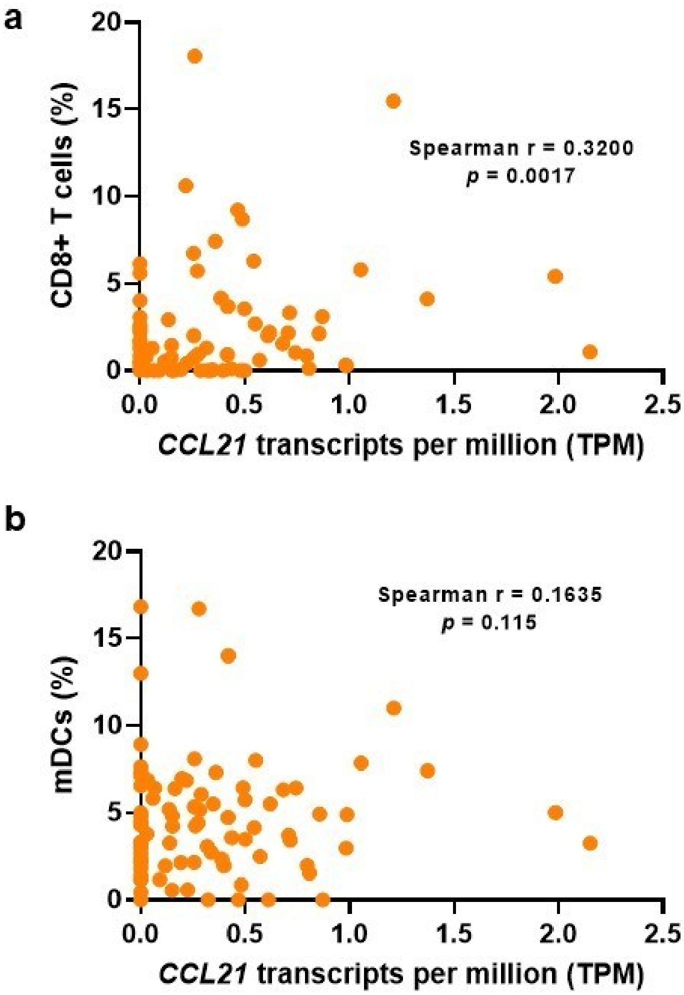

Supplementary Fig. 5. Correlation of *CCL21* mRNA with computationally inferred immune cell infiltrate in MBM. a, Spearman rank correlation of CD8+ T cell percentage with *CCL21* mRNA transcripts in MBM. **b,** Spearman rank correlation of mDC immune cell percentage with *CCL21* mRNA transcripts.
