# Supplementary material for: Multi-omic profiling reveals discrepant immunogenic properties and a unique tumor microenvironment among melanoma brain metastases
Source: NPJ Precis Oncol. 2023 Nov 14;7:120. doi: 10.1038/s41698-023-00471-z (PMC10646102; doi:10.1038/s41698-023-00471-z)
Supplement: Supplementary file 5 — REPORTING SUMMARY [file 41698_2023_471_MOESM5_ESM.pdf]

nature portfolio

Corresponding author(s): Gino K. In

Last updated by author(s): Jul 31, 2023

Reporting Summary

Nature Portfolio wishes to improve the reproducibility of the work that we publish. This form provides structure for consistency and transparency in reporting. For further information on Nature Portfolio policies, see our Editorial Policies and the Editorial Policy Checklist.

Statistics

For all statistical analyses, confirm that the following items are present in the figure legend, table legend, main text, or Methods section.

n/a	Confirmed	
☐	☒	The exact sample size (*n*) for each experimental group/condition, given as a discrete number and unit of measurement
☐	☒	A statement on whether measurements were taken from distinct samples or whether the same sample was measured repeatedly
☐	☒	The statistical test(s) used AND whether they are one- or two-sided<br>*Only common tests should be described solely by name; describe more complex techniques in the Methods section.*
☒	☐	A description of all covariates tested
☐	☒	A description of any assumptions or corrections, such as tests of normality and adjustment for multiple comparisons
☐	☒	A full description of the statistical parameters including central tendency (e.g. means) or other basic estimates (e.g. regression coefficient) AND variation (e.g. standard deviation) or associated estimates of uncertainty (e.g. confidence intervals)
☐	☒	For null hypothesis testing, the test statistic (e.g. *F*, *t*, *r*) with confidence intervals, effect sizes, degrees of freedom and *P* value noted<br>*Give P values as exact values whenever suitable.*
☒	☐	For Bayesian analysis, information on the choice of priors and Markov chain Monte Carlo settings
☒	☐	For hierarchical and complex designs, identification of the appropriate level for tests and full reporting of outcomes
☐	☒	Estimates of effect sizes (e.g. Cohen's *d*, Pearson's *r*), indicating how they were calculated

Our web collection on statistics for biologists contains articles on many of the points above.

Software and code

Policy information about availability of computer code

Data collection	No software used.
Data analysis	Standard statistical methods in R version 4 and GraphPad Prism version 9 were employed to analyze the data.

For manuscripts utilizing custom algorithms or software that are central to the research but not yet described in published literature, software must be made available to editors and reviewers. We strongly encourage code deposition in a community repository (e.g. GitHub). See the Nature Portfolio guidelines for submitting code & software for further information.

Data

Policy information about availability of data

All manuscripts must include a data availability statement. This statement should provide the following information, where applicable:

- Accession codes, unique identifiers, or web links for publicly available datasets
- A description of any restrictions on data availability
- For clinical datasets or third party data, please ensure that the statement adheres to our policy

The datasets generated and/or analyzed during the current study are available for replication and verification purposes from the corresponding author on reasonable request. The de-identified DNA and RNA sequencing data are owned by Caris Life Sciences and cannot be publicly shared due to the data usage agreement signed by Dr. Heinz-Josef Lenz at Keck School of Medicine of USC. Qualified researchers can apply for access to these data by contacting Joanne Xiu, PhD

at jxiu@carisls.com, submitting a brief proposal, and signing a data usage agreement. The summarized sequencing data are available within the Supplemental Materials for this paper and at https://figshare.com/s/4f0dbb3aaf58d17f4615.

Research involving human participants, their data, or biological material

Policy information about studies with human participants or human data. See also policy information about sex, gender (identity/presentation), and sexual orientation and race, ethnicity and racism.

Reporting on sex and gender	The breakdown of M vs. F sex among the cohort was reported. No additional sex-specific analyses were performed.
Reporting on race, ethnicity, or other socially relevant groupings	N/A. No race or ethnicity information was available.
Population characteristics	Limited demographic or treatment information was available for the cohort.
Recruitment	Patient samples were selected for inclusion according to availability in the Caris database.
Ethics oversight	N/A. This study is considered IRB exempt.

Note that full information on the approval of the study protocol must also be provided in the manuscript.

Field-specific reporting

Please select the one below that is the best fit for your research. If you are not sure, read the appropriate sections before making your selection.

☒ Life sciences ☐ Behavioural & social sciences ☐ Ecological, evolutionary & environmental sciences

For a reference copy of the document with all sections, see nature.com/documents/nr-reporting-summary-flat.pdf

Life sciences study design

All studies must disclose on these points even when the disclosure is negative.

Sample size	Patient samples were selected for inclusion in the study according to availability in the Caris CODEai database. A total of 1,314 samples were included, which is to our knowledge the largest melanoma cohort to date analyzed according to primary or metastatic location.
Data exclusions	N/A. No patients were excluded.
Replication	Due to the nature of the study, a secondary validation cohort was not analyzed. Other studies have been performed in smaller cohorts that are supportive of our findings.
Randomization	N/A. Randomization was not performed since grouping was performed based on tumor-location. This is a retrospective study.
Blinding	N/A. Blinding was not performed. This is a retrospective study.

Reporting for specific materials, systems and methods

We require information from authors about some types of materials, experimental systems and methods used in many studies. Here, indicate whether each material, system or method listed is relevant to your study. If you are not sure if a list item applies to your research, read the appropriate section before selecting a response.

Materials & experimental systems

n/a	Involved in the study	
☐	☒	Antibodies
☒	☐	Eukaryotic cell lines
☒	☐	Palaeontology and archaeology
☒	☐	Animals and other organisms
☒	☐	Clinical data
☒	☐	Dual use research of concern
☒	☐	Plants

Methods

n/a	Involved in the study	
☒	☐	ChIP-seq
☒	☐	Flow cytometry
☒	☐	MRI-based neuroimaging

Antibodies

Antibodies used	The primary antibody used for PD-L1 was clone SP142 (Spring Biosciences).
Validation	Caris is a CLIA/CAP certified laboratory and all IHC results were validated per CLIA/CAP requirements, as stated in the methods.
